# Antimicrobial Resistance and Genetic Characteristics of Ciprofloxacin-Resistant *Escherichia coli* from Suckling and Weaned Piglet Housing Areas in Korea

**DOI:** 10.3390/microorganisms14102247

**Published:** 2026-10-03

**Authors:** Oyoung Kwon, Da-Hye Ryu, Hyun-Jung Ahn, Su-Jin Choe, Min-Gyu Kim, You-Kyung Go, Su-Bin Kim, Soochong Kim, Seung-Hun Lee, Kyung-Hyo Do, Kwang-Won Seo

**Affiliations:** 1 College of Veterinary Medicine, Chungbuk National University, Cheongju 28644, Republic of Korea; 2 Department of Companion Animal Health, Daegu Haany University, Gyeongsan 38610, Republic of Korea

**Keywords:** *Escherichia coli*, fluoroquinolone resistance, swine production stage, quinolone resistance-determining region, plasmid-mediated quinolone resistance

## Abstract

In swine production systems, antimicrobial exposure and management conditions vary across production stages, particularly during the transition from suckling to weaning, which may influence the selection and persistence of antimicrobial-resistant bacteria. We compared the antimicrobial resistance patterns of 70 *Escherichia coli* isolates obtained from suckling and weaned piglet housing areas (35 isolates each) on 35 farms in Korea and further characterized the genetic features of the 40 ciprofloxacin (CIP)-resistant isolates. Isolates showed high rates of resistance to several antimicrobials, including streptomycin (88.6%) and ampicillin (85.7%). In an exploratory comparison, CIP resistance was higher in isolates from weaned piglet housing areas (71.4%) than in those from suckling piglet housing areas (42.9%; unadjusted two-sided exact *p* = 0.04139, McNemar’s test). Mutations in the quinolone resistance-determining regions (QRDRs) were detected in 27 of the 40 CIP-resistant isolates (67.5%), predominantly *gyrA* S83L/D87N and *parC* S80I substitutions. Plasmid-mediated quinolone resistance (PMQR) determinants were detected in nine isolates; *qnrD* was the most common gene (6/9, 66.7%). Multilocus sequence typing analysis of the nine PMQR-positive isolates identified multiple sequence types; ST5229 was the most common sequence type (5/9, 55.6%) and was detected in both housing-area groups. The higher CIP resistance observed in isolates from weaned piglet housing areas indicates a production-stage-associated difference in FQ resistance during early pig production.

## 1. Introduction

Fluoroquinolones (FQs) are widely used in human and veterinary medicine because of their broad-spectrum activity and high efficacy against Gram-negative bacteria [1]. Quinolones, including FQs, are classified as highest priority critically important antimicrobials in the 2024 WHO List of Medically Important Antimicrobials [2]. However, the increasing use of FQs in livestock production has contributed to the emergence and dissemination of FQ-resistant *Escherichia coli*, raising concerns regarding animal health, food safety, and public health [3,4].

FQ resistance in *E. coli* primarily arises through chromosomal mutations in the quinolone resistance-determining regions (QRDRs) of genes encoding DNA gyrase (*gyrA*) and topoisomerase IV (*parC*) [5]. These mutations reduce drug binding to the target enzymes and are frequently associated with high levels of resistance [5]. In addition, plasmid-mediated quinolone resistance (PMQR) determinants, including *qnr* variants, *aac(6′)-Ib-cr*, and efflux pump genes such as *qepA*, contribute to the dissemination of resistance [6]. Although PMQR genes typically confer low-level resistance, they facilitate the selection of additional chromosomal mutations and accelerate the spread of FQ resistance among bacterial populations via horizontal gene transfer [6].

Swine production systems are characterized by multiple production stages, including suckling and weaning, stages each associated with distinct physiological and management conditions [7]. During weaning, piglets experience substantial physiological stress, dietary transitions, and declining maternal immunity, which increase their susceptibility to infectious diseases [8]. Consequently, antimicrobial use intensifies during this stage, potentially promoting the selection and persistence of antimicrobial-resistant bacteria [9,10]. In Korean swine production systems, the post-weaning period has been reported to be an important stage for antimicrobial treatment because piglets are particularly susceptible to enteric and respiratory diseases [11,12]. Antimicrobial exposure during this stage may promote the selection and persistence of antimicrobial resistant bacteria [9]. Once resistant bacteria emerge on farms, they can be disseminated through multiple routes, including direct animal contact, environmental contamination, feed and water, and farm personnel [9].

Previous Korean studies separately documented antimicrobial-resistant *E. coli* in suckling piglets with colibacillosis and in diarrheic weaned piglets [13,14]. More recently, a stage-stratified study conducted on Korean pig farms reported that ciprofloxacin (CIP) resistance was higher in isolates from weaned piglet housing areas (66.7%) than in those from suckling piglet housing areas (42.9%) [1]. Although this study integrated phenotypic and molecular analyses across broader swine production stages, studies focused specifically on the early suckling-to-weaning transition with balanced representation of both stages across farms remain limited. Consequently, how FQ-resistance phenotypes, resistance determinants, and clonal backgrounds differ between these two early production stages remains insufficiently understood. Although antimicrobial susceptibility was evaluated across the full *E. coli* collection, molecular characterization focused on the FQ-resistant subset. This focus was chosen because FQs are critically important antimicrobials and FQ resistance in *E. coli* involves both chromosomal QRDR mutations and transferable PMQR determinants, providing a useful framework for investigating the persistence and dissemination of resistance across production stages [1]. Therefore, we compared the antimicrobial resistance profiles of *E. coli* isolates from suckling and weaned piglet housing areas in Korea and further characterized the molecular and clonal features of the FQ-resistant subset. We hypothesized that *E. coli* isolates from weaned piglet housing areas would exhibit a higher frequency of CIP resistance than those from suckling piglet housing areas and that shared resistance determinants and clonal backgrounds would be detected across both production stages.

## 2. Materials and Methods

### 2.1. Ethics Approval

Fecal samples were collected from the floors of suckling and weaned piglet housing areas without invasive procedures or experimental interventions involving animals. The study was exempt from formal ethical review, as confirmed by the Institutional Animal Care and Use Committee of Chungbuk National University. Permission for sample collection and research use was obtained from the participating farm owners.

### 2.2. Bacterial Isolates

Fecal samples were collected from pen floors on 35 pig farms in South Korea between March 2022 and February 2023. At each farm, samples from the suckling and weaned piglet housing areas were collected on the same day during the same farm visit. Only farms housing both suckling and weaned piglets were included in the study to enable stage-specific comparisons. At each farm, fecal material was collected using sterile gauze swabs from 15 sampling points on pen floors in each of the suckling and weaned piglet housing areas, totaling 30 sampling points per farm [15]. Samples collected within each production stage were pooled separately to generate one stage-specific composite fecal sample for the suckling piglet housing area and one for the weaned piglet housing area. Approximately 10 g of fecal material was collected from each sampling point. Samples were placed in sterile containers, transported to the laboratory within 24 h under chilled conditions using ice packs, and processed upon arrival. For bacterial isolation, each stage-specific composite fecal sample was streaked onto MacConkey agar plates (BD Biosciences, Franklin Lakes, NJ, USA) and incubated overnight at 37 °C. Presumptive *E. coli* colonies showing typical pink colonies on MacConkey agar were selected and confirmed using PCR, as previously described [15]. From each stage-specific composite fecal sample, one isolate was randomly selected from the PCR-confirmed *E. coli* colonies before antimicrobial susceptibility testing, irrespective of its antimicrobial susceptibility profile. This approach ensured equal representation across farms and production stages, with each farm contributing one isolate from the suckling piglet housing area and one from the weaned piglet housing area. This yielded 70 *E. coli* isolates, comprising 35 isolates from each production stage. The farms, source samples, and isolates included in this study did not overlap with those in our previous publications. The study size was based on investigator judgment, without a prespecified target number of farms or a formal a priori sample-size or power calculation. Thus, the 70 isolates were not selected on the basis of CIP resistance.

### 2.3. Antimicrobial Susceptibility Test

The antimicrobial susceptibilities of all *E. coli* isolates were evaluated using the disk diffusion method, and the results were interpreted according to the Clinical and Laboratory Standards Institute (CLSI) VET01S guidelines [16]. The antimicrobial agents tested were ampicillin (10 μg), amoxicillin–clavulanic acid (20/10 μg), piperacillin–tazobactam (100/10 μg), cefazolin (30 μg), cefuroxime (30 μg), cefoxitin (30 μg), ceftiofur (30 μg), cefotaxime (30 μg), ceftazidime (30 μg), cefepime (30 μg), gentamicin (10 μg), streptomycin (10 μg), kanamycin (30 μg), oxytetracycline (30 μg), tetracycline (30 μg), tigecycline (15 μg), florfenicol (30 μg), chloramphenicol (30 μg), nalidixic acid (30 μg), ciprofloxacin (CIP, 5 μg), sulfisoxazole (250 μg), trimethoprim–sulfamethoxazole (1.25/23.75 μg), and meropenem (10 μg). Resistance frequencies were calculated as the percentage of resistant isolates among all isolates tested for each antimicrobial. Isolates with intermediate susceptibility were retained in the intermediate category and included in the denominator, but not in the resistant-isolate count. Multidrug resistance (MDR) was operationally defined as resistance to at least one antimicrobial agent in three or more antimicrobial classes, based on disk diffusion results. Intermediate results were not counted as resistant for MDR classification. The minimum inhibitory concentrations (MICs) of CIP, norfloxacin (NOR), and enrofloxacin (ENR) were determined for all 40 CIP-resistant isolates using the broth microdilution method with twofold serial dilutions ranging from 0.125 to 256 μg/mL for each antimicrobial, in accordance with CLSI recommendations [14]. MIC50 and MIC90 were determined from the cumulative MIC distributions as the lowest concentrations inhibiting at least 50% and 90% of the isolates, respectively. Calculations included all 40 CIP-resistant isolates, with production-stage-specific values determined separately for the 15 isolates from suckling piglet housing areas and the 25 isolates from weaned piglet housing areas. MICs exceeding 256 μg/mL were retained as >256 μg/mL without substitution by a numerical value. When either percentile endpoint exceeded the highest tested concentration, it was reported as >256 μg/mL. The term “high MICs” is used descriptively to refer to the measured MIC values and does not denote a separate susceptibility category or a predefined threshold. CIP, NOR, and ENR were selected as representative FQs of human and veterinary relevance, allowing comparison of resistance levels across related agents within the same antimicrobial class. CIP and NOR represent human-use FQs, whereas ENR is a veterinary FQ relevant to swine production. The MIC thresholds used in this study to classify isolates as susceptible, intermediate, and resistant were ≤0.25, 0.5, and ≥1 μg/mL for CIP; ≤4, 8, and ≥16 μg/mL for NOR; and ≤0.06, 0.12–0.25, and ≥0.5 μg/mL for ENR, respectively [16]. The reference strain *E. coli* ATCC 25922 was used for quality control. Isolates with a CIP inhibition zone diameter of ≤21 mm using a 5 μg disk were classified as CIP-resistant according to CLSI VET01S [16]. Of the 70 *E. coli* isolates tested, 40 met this definition and constituted the CIP-resistant subset for subsequent FQ-specific analyses.

### 2.4. Detection of Antimicrobial Resistance Genes and Analysis of Mutations in QRDRs

Among the 70 *E. coli* isolates, the 40 isolates classified as CIP-resistant were selected for subsequent molecular analyses, including QRDR and PMQR characterization. All PCR assays included an initial denaturation step at 95 °C for 15 min, followed by 30 amplification cycles. The QRDRs of *gyrA* and *parC* were amplified using the forward and reverse primers 5′-AAATCTGCCCGTGTCGTTGGT-3′ and 5′-GCCATACCTACGGCGATACC-3′ for *gyrA* (344 bp), and 5′-AAACCTGTTCAGCGCCGCATT-3′ and 5′-GTGGTGCCGTTAAGCAAA-3′ for *parC* (395 bp). Annealing temperatures were 55 °C and 59 °C, respectively. The amplified products were purified and subjected to Sanger sequencing by a commercial sequencing service provider (Macrogen Inc., Seoul, Republic of Korea). The resulting *gyrA* and *parC* nucleotide sequences were compared with the corresponding reference sequences of *E. coli* K-12 substr. MG1655 using the NCBI web-based BLASTn service to identify QRDR mutations.

All 40 CIP-resistant isolates were screened for PMQR determinants, including *qnrA*, *qnrB*, *qnrC*, *qnrD*, *qnrS*, *aac(6′)-Ib-cr*, and *qepA*, using PCR followed by sequence confirmation [14]. The forward and reverse primer sequences (5′–3′), expected amplicon sizes, and annealing temperatures were as follows: *qnrA*, TCAGCAAGAGGATTTCTCA/GGCAGCACTATTACTCCCA (627 bp; 48 °C); *qnrB*, CGACCTGAGCGGCACTGAAT/TGAGCAACGATGCCTGGTAG (515 bp; 58 °C); *qnrD*, CGAGATCAATTTACGGGGAATA/AACAAGCTGAAGCGCCTG (582 bp; 53 °C); *qnrS*, ACCTTCACCGCTTGCACATT/CCAGTGCTTCGAGAATCAGT (571 bp; 58 °C); *qepA*, CGTGTTGCTGGAGTTCTTC/CTGCAGGTACTGCGTCATG (403 bp; 51 °C); and *aac(6′)-Ib*, TGACCTTGCGATGCTCTATG/TTAGGCATCACTGCGTGTTC (508 bp; 56 °C). Isolates carrying PMQR genes were further analyzed for additional antimicrobial resistance determinants. These included β-lactamase genes (*blaTEM*, *blaSHV*, *blaOXA*, and *blaCTX-M* families), tetracycline resistance genes (*tetA*, *tetB*, and *tetC*), chloramphenicol resistance genes (*cmlA* and *catA1*), sulfonamide resistance genes (*sul1* and *sul2*), and aminoglycoside resistance genes (*aac(3)-II* and *ant(2″)-I*).

The presence of class I and class II integrons (*intI1* and *intI2*) was also investigated. The forward and reverse primer sequences (5′–3′) were GCCTTGCTGTTCTTCTACGG/GATGCCTGCTTGTTCTACGG for *intI1* and CACGGATATGCGACAAAAAGGT/GTAGCAAACGAGTGACGAAATG for *intI2*. Amplification cycles comprised denaturation at 94 °C for 30 s, annealing at 58 °C for 30 s, and extension at 72 °C for 2 min. Gene cassettes associated with integron-positive isolates were amplified, purified, and sequenced, as previously described. Gene cassette sequences were analyzed using BLASTn against the NCBI nucleotide collection (nt) database with default search parameters.

### 2.5. Multilocus Sequence Typing and Analysis of MLST-Based Relationships

To characterize the clonal backgrounds of PMQR-positive isolates, multilocus sequence typing (MLST) was performed on all nine PMQR-positive *E. coli* isolates. The internal fragments of seven housekeeping genes (*adk*, *fumC*, *gyrB*, *icd*, *mdh*, *purA*, and *recA*) were amplified and sequenced using the standard *E. coli* MLST scheme. Allele numbers and sequence types (STs) were assigned by comparison with the PubMLST database for *E. coli* [17]. The nucleotide sequences of the seven housekeeping genes were concatenated and aligned using ClustalW with default parameters in MEGA X [1]. Evolutionary distances were estimated using the maximum composite likelihood method, including both transitions and transversions, with gamma-distributed rate variation among sites (shape parameter = 1.0) and homogeneous substitution patterns among lineages. Gaps and missing data were handled by pairwise deletion. A neighbor-joining tree with 1000 bootstrap replicates was constructed to provide a descriptive visualization of sequence differences across the MLST loci among the nine PMQR-positive isolates. 

### 2.6. Statistical Analysis

All statistical analyses were performed using SPSS Statistics software (version 27.0; SPSS, Inc., Chicago, IL, USA). Farm was considered the primary sampling unit, with one isolate from each production stage serving as the observational unit for resistance assessment. Differences in antimicrobial resistance rates between isolates from suckling and weaned piglet housing areas were evaluated using McNemar’s test, with farm as the pairing unit to account for within-farm correlation. The antimicrobial comparisons were exploratory, and CIP was not prespecified as the primary comparison. No adjustment for multiple testing was applied; therefore, the reported *p*-values are unadjusted, and findings with *p* < 0.05 are interpreted as hypothesis-generating, with an acknowledged risk of false-positive findings.

## 3. Results

### 3.1. Antimicrobial Resistance Profiles of E. coli from Suckling and Weaned Piglet Housing Area

**Figure 1:**
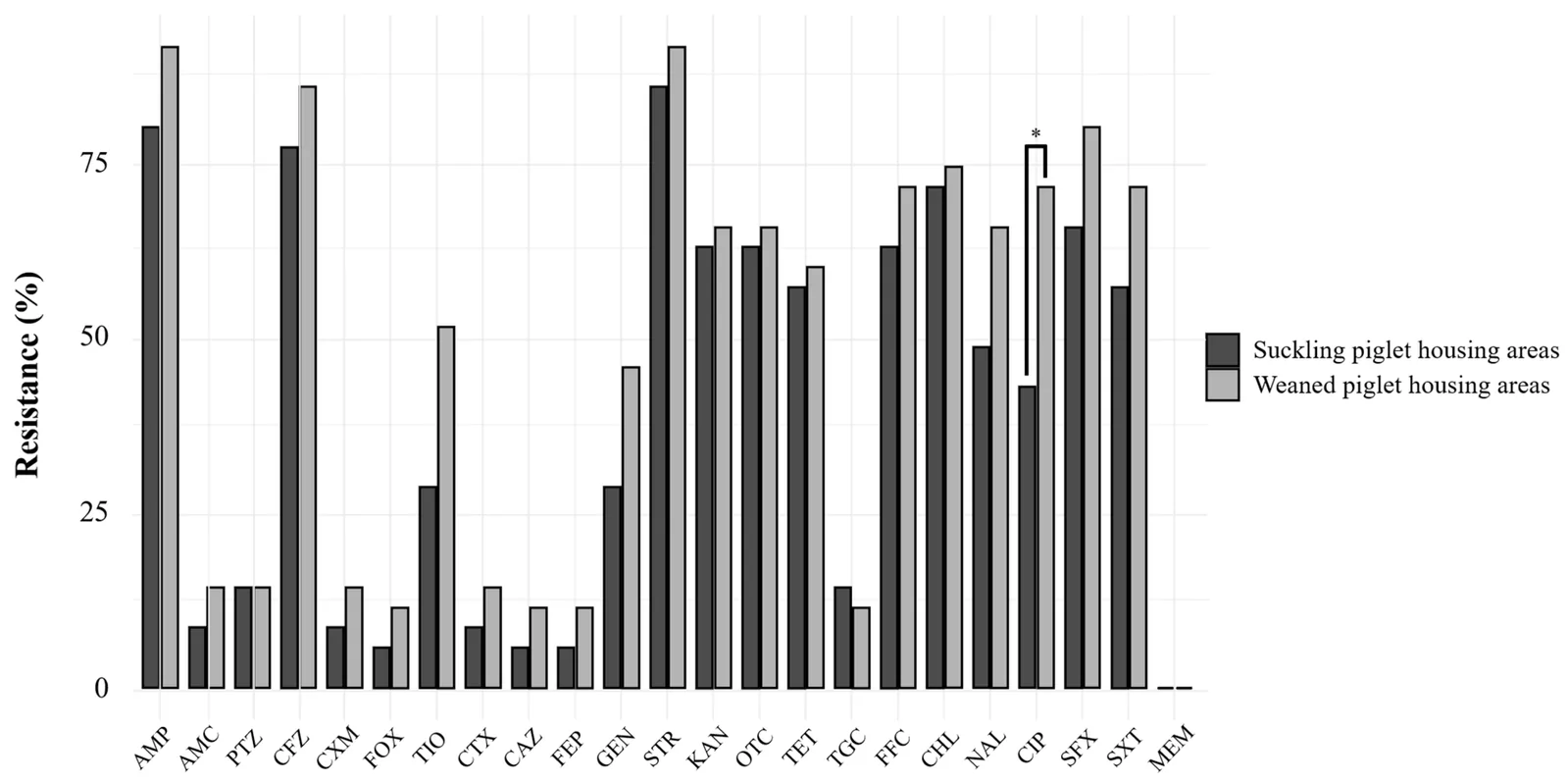
Antimicrobial resistance of *Escherichia coli* from suckling and weaned piglet housing areas in Korea. Dark gray bars indicate isolates from suckling piglet housing areas, and light gray bars indicate isolates from weaned piglet housing areas. An asterisk indicates a statistically significant difference between isolates from suckling and weaned piglet housing areas (*p* < 0.05). AMP: ampicillin; AMC: amoxicillin–clavulanate; PTZ: piperacillin–tazobactam; CFZ: cefazolin; CXM: cefuroxime; FOX: cefoxitin; TIO: ceftiofur; CTX: cefotaxime; CAZ: ceftazidime; FEP: cefepime; GEN: gentamicin; STR: streptomycin; KAN: kanamycin; OTC: oxytetracycline; TET: tetracycline; TGC: tigecycline; FFC: florfenicol; CHL: chloramphenicol; NAL: nalidixic acid; CIP: ciprofloxacin; SFX: sulfisoxazole; SXT: trimethoprim–sulfamethoxazole; MEM: meropenem.

Antimicrobial resistance patterns of *E. coli* isolates from suckling and weaned piglet housing area are shown in Figure 1. Of the 70 *E. coli* isolates selected before antimicrobial susceptibility testing and irrespective of their susceptibility profiles, 40 (57.1%) were classified as CIP-resistant. All 40 CIP-resistant isolates were included in the subsequent FQ-specific analyses; no subset was selected. Overall, high resistance rates were observed to several antimicrobial agents in both groups. The highest resistance rates were detected against streptomycin (88.6%) and ampicillin (85.7%), followed by cefazolin (81.4%), chloramphenicol (72.9%), and sulfisoxazole (72.9%). Moderate resistance was observed against tetracyclines and phenicols including oxytetracycline (64.3%), tetracycline (58.6%), and florfenicol (67.1%). In contrast, resistance to β-lactam/β-lactamase inhibitor combinations and higher-generation cephalosporins remained relatively low. Resistance to meropenem was not observed. When resistance rates were compared between housing-area groups, isolates from weaned piglet housing areas generally exhibited higher frequencies of resistance to several antimicrobials than those from suckling piglet housing areas. However, CIP resistance was significantly higher in isolates from weaned piglet housing areas (71.4%) than in those from suckling piglet housing areas (42.9%) (matched-pair odds ratio = 3.00; exact 95% CI: 1.036–10.551; two-sided exact *p* = 0.04139).

### 3.2. QRDR Mutations, PMQR Genes, and FQ MICs of CIP-Resistant E. coli from Suckling and Weaned Piglet Housing Areas

The QRDR mutations, PMQR genes, and FQ MIC of the CIP-resistant *E. coli* isolates are summarized in Table 1. All 40 CIP-resistant isolates were analyzed for QRDR mutations. Mutations were detected in 27 isolates (67.5%), whereas no mutations were detected in the analyzed regions of *gyrA* and *parC* in the remaining 13 isolates (32.5%). Among the 27 isolates with detected QRDR mutations, double mutations in *gyrA* (S83L and D87N) were observed in 18 isolates (66.7%), whereas a substitution in *parC* (S80I) was detected in 22 isolates (81.5%). Combined mutation patterns of *gyrA* S83L/D87N and *parC* S80I were identified in 16 isolates (59.3%). Combined mutation patterns of *gyrA* S83L/D87N and *parC* S80I were detected in seven isolates from suckling piglet housing areas and nine isolates from weaned piglet housing areas.

PMQR determinants were detected in nine isolates (4 from suckling piglet housing areas and 5 from weaned piglet housing areas). Among these, *qnrD* was the most frequently identified gene and was detected in six isolates (3 each from suckling and weaned piglet housing areas). Other PMQR determinants included *qnrB* and *qnrS*, each detected in one isolate, whereas *qnrS* with *qepA* and *qnrD* with *aac(6′)-Ib-cr* were identified in one isolate each.

The MIC distribution of FQs showed high resistance among the isolates. Overall, the MIC_50_ and MIC_90_ values were 32 and 128 μg/mL for CIP, 256 and >256 μg/mL for ENR, and 256 and 256 μg/mL for NOR, respectively. When stratified by housing area groups, isolates from suckling piglet housing areas showed MIC_50_/MIC_90_ values of 16/64 μg/mL for CIP, 256/>256 μg/mL for ENR, and 256/256 μg/mL for NOR. In contrast, isolates from weaned piglet housing areas showed higher CIP MIC values (MIC_50_/MIC_90_ = 32/128 μg/mL) and ENR MIC values (MIC_50_/MIC_90_ >256/>256 μg/mL), whereas NOR MIC values remained 256/256 μg/mL.

### 3.3. MLST-Based Relationships Among PMQR-Positive E. coli Isolates

**Figure 2:**
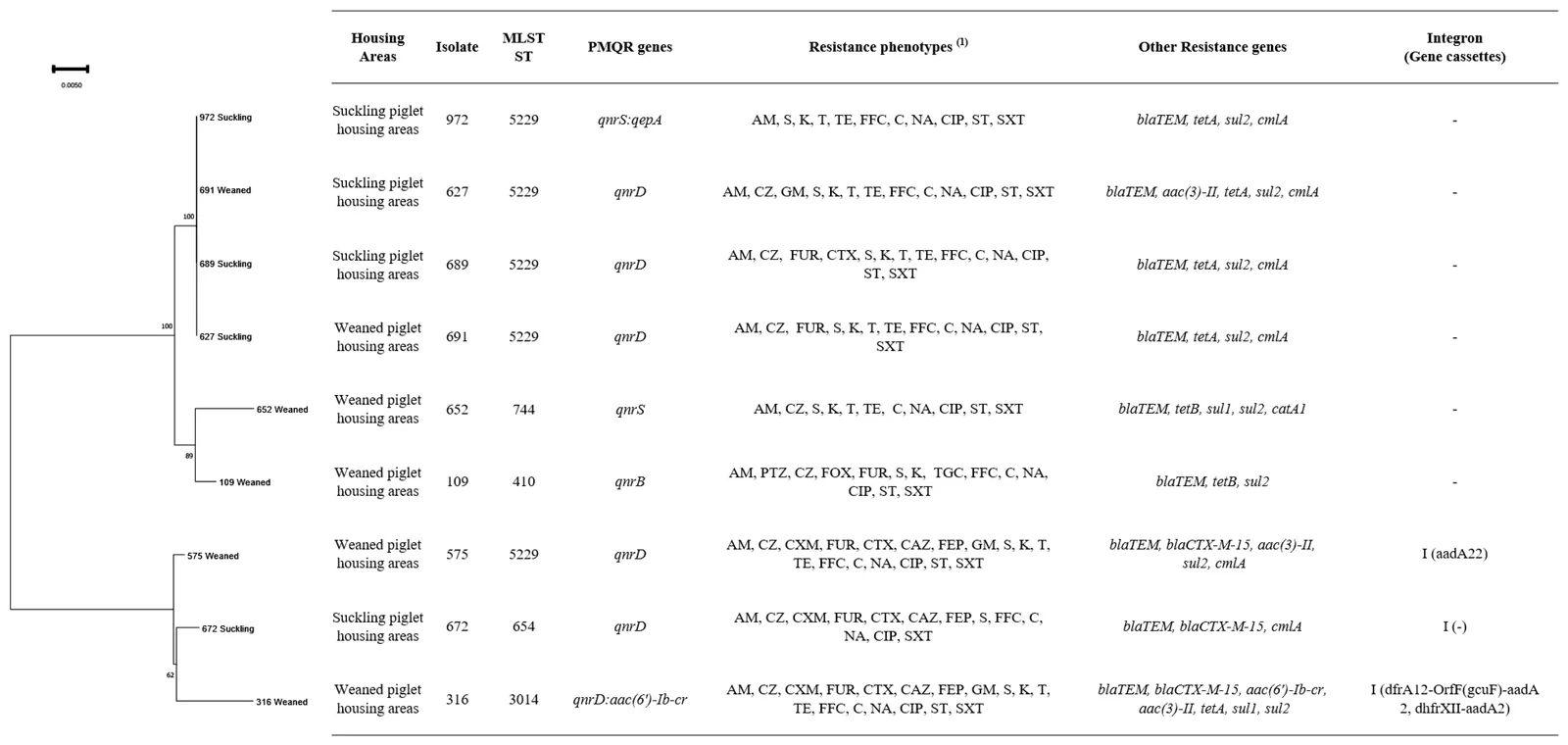
Neighbor-joining tree based on concatenated sequences of seven MLST loci, illustrating MLST-based relationships among nine PMQR-positive *Escherichia coli* isolates from suckling and weaned piglet housing areas in Korea. Bootstrap values based on 1000 replicates are shown at branch nodes. Scale bar indicates nucleotide substitutions per site. ^(1)^ AM: ampicillin; C: chloramphenicol; CAZ: ceftazidime; CIP: ciprofloxacin; CTX: cefotaxime; CXM: cefuroxime; CZ: cefazolin; FEP: cefepime; FFC: florfenicol; FOX: cefoxitin; FUR: ceftiofur; GM: gentamicin; K: kanamycin; NA: nalidixic acid; PTZ: piperacillin–tazobactam; S: streptomycin; ST: sulfisoxazole; SXT: trimethoprim-sulfamethoxazole; T: oxytetracycline; TE: tetracycline; TGC: tigecycline.

The MLST-based relationships among the nine PMQR-positive *E. coli* isolates from suckling and weaned piglet housing areas are shown in Figure 2. MLST analysis identified five STs, with ST5229 (ST101 Cplx) as the predominant lineage, accounting for five of the nine isolates (55.6%). The remaining isolates belonged to ST744, ST410 (ST23 Cplx), ST654, and ST3014, each represented by one isolate (11.1%). The five ST5229 isolates included three from suckling piglet housing areas and two from weaned piglet housing areas.

The phylogenetic tree showed that isolates from suckling and weaned piglet housing areas were distributed across multiple branches rather than forming completely separate stage-specific groups. Particularly, isolates belonging to ST5229 clustered closely together despite originating from both suckling and weaned piglet housing areas. Other PMQR-positive isolates, including those assigned to ST744, ST410, ST654, and ST3014, formed branches distinct from the ST5229 cluster.

All nine PMQR-positive isolates showed a multidrug resistance phenotype. Additional resistance genes were variably distributed among isolates. *blaTEM* was detected in all nine isolates, whereas *blaCTX-M-15* was detected in only three isolates (33.3%). Class 1 integrons were identified in three isolates (33.3%), including one carrying aadA22, one with an empty integron structure, and one harboring *dfrA12-OrfF(gcuF)-aadA2* and *dhfrXII-aadA2* gene cassettes.

## 4. Discussion

In this study, isolates from weaned piglet housing areas tended to show higher resistance frequencies to several antimicrobials than those from suckling piglet housing areas, with a nominally significant difference observed only for CIP. Although physiological and management changes occur during weaning, including dietary transition, environmental stress, and declining maternal immunity, their contributions to the observed difference in CIP resistance could not be determined in this study [7,8]. Our findings are consistent with previous stage-stratified observations in Korean pig farms, in which CIP resistance was higher among isolates from weaned piglet housing areas than among isolates from other production-stage groups, supporting a production-stage-associated difference in FQ resistance in swine-associated *E. coli* [1]. In contrast, resistance to β-lactam/β-lactamase inhibitor combinations and higher-generation cephalosporins remained relatively low, and no meropenem resistance was detected [18].

QRDR mutations were detected in 27 of the 40 CIP-resistant isolates (67.5%), indicating that these mutations were common among the resistant isolates. However, targeted PCR/Sanger sequencing did not comprehensively assess efflux mechanisms, mutations outside the analyzed regions, or other resistance determinants; therefore, their relative contributions to FQ resistance could not be determined [6,14]. The frequent detection of the combined *gyrA* S83L/D87N and *parC* S80I patterns is consistent with previous reports showing that the sequential accumulation of mutations in DNA gyrase and topoisomerase IV is strongly associated with high-level resistance to FQs in swine-associated *E. coli* [6,14].

PMQR genes were detected in nine of the 40 CIP-resistant isolates, including isolates from both suckling and weaned piglet housing areas. Their detection establishes their presence in these isolates but does not demonstrate plasmid localization, mobility, or horizontal transfer [14,19,20]. Among these determinants, *qnrD* was the most frequently detected, whereas *qnrB*, *qnrS*, *qnrS* + *qepA*, and *qnrD* + *aac(6′)-Ib-cr* were detected sporadically. This distribution is broadly consistent with previous swine studies, in which *qnrD* and *qnrS* were among the major PMQR genes detected in porcine *E. coli* [1,14,21].

The MIC results further support this interpretation. Overall, the isolates showed high MICs for CIP, ENR, and NOR, indicating that the resistant populations in both age groups had already acquired a substantial degree of FQ resistance. Notably, the high MIC values observed in many isolates carrying the common *gyrA*/*parC* mutation combination were consistent with the established relationship between double QRDR mutations and high FQ MICs [14,22,23]. Additionally, the presence of PMQR-positive isolates with variable mutation profiles suggests that resistance expression is not determined by a single mechanism, but rather by the interaction of chromosomal mutations and plasmid-mediated determinants. This interpretation is supported by previous Korean and international studies showing that PMQR genes can enhance or maintain resistance phenotypes even when the full set of canonical QRDR mutations is absent [1,14]. Collectively, these findings demonstrate the frequent occurrence of QRDR mutations and the presence of PMQR determinants among CIP-resistant *E. coli* isolates from both production stages. The broadly similar molecular patterns detected in both age groups indicate that related resistance determinants were present in isolates from both suckling and weaned piglet housing areas. Previous studies have frequently identified weaning as a critical period for antimicrobial resistance selection because of increased disease susceptibility and antimicrobial exposure [24,25,26]. However, the present findings suggest that a substantial proportion of the resistance framework may already be established before weaning. The detection of similar QRDR mutation patterns and PMQR-positive isolates from suckling piglet housing areas shows that these resistance features were also present in that housing-area group. Rather, highly resistant strains or resistance determinants were detected in isolates from both suckling and weaned piglet housing areas. This observation expands current understanding of FQ resistance ecology in pig production by suggesting that resistance amplification during weaning may occur on the basis of pre-existing resistant populations rather than through de novo emergence after weaning. Among the nine PMQR-positive isolates subjected to MLST, ST5229 was detected in isolates from both suckling and weaned piglet housing areas [1].

The additional detection of ST744, ST410, ST654, and ST3014 in separate branches further suggests that PMQR-positive isolates from the piglet housing areas are genetically heterogeneous and not represented by a single clonal lineage [27]. The detection of PMQR genes in isolates belonging to different sequence types does not establish their plasmid context or transfer between isolates [28].

Notably, all PMQR-positive isolates exhibited multidrug-resistant phenotypes and universally carried *blaTEM*, whereas *blaCTX-M-15* and class 1 integrons were detected in a subset of isolates. A similar pattern was observed in our previous studies, in which PMQR-harboring isolates frequently showed multidrug resistance, widespread *blaTEM* carriage, and occasional associations with class 1 integrons and diverse gene cassettes [1]. Therefore, the present findings support the view that PMQR-positive *E. coli* in suckling and weaned piglet housing areas should be regarded as components of a broader multidrug resistance framework rather than as isolates carrying only quinolone-associated determinants. In practical terms, this means that antimicrobial resistance control strategies should not focus only on reducing the selection of a single clone but also on limiting the maintenance and spread of multidrug resistance platforms across interconnected production stages [29]. These findings generate the hypothesis that sampling fecal material from suckling and weaned piglet housing areas could provide additional information on production-stage differences in antimicrobial resistance. However, this study did not evaluate surveillance strategies or estimate population-level AMR prevalence and therefore cannot establish that surveillance restricted to later production stages underestimates such prevalence. The added value of early-life sampling requires evaluation in larger studies with representative sampling across production stages.

CIP-resistant *E. coli* and shared resistance determinants and sequence types were detected in isolates from both suckling and weaned piglet housing areas, with higher CIP resistance among isolates from weaned piglet housing areas. These findings indicate production-stage-associated differences but do not establish that resistant strains or resistance determinants persisted through the suckling-to-weaning transition, because the same animals were not followed longitudinally. QRDR mutations, PMQR genes, and additional resistance determinants were detected together in some isolates, but their physical linkage and functional interactions were not investigated [14,30,31]. These findings suggest that CIP-resistant *E. coli* with diverse genetic backgrounds are present at both suckling and weaned stages, with higher CIP resistance observed among isolates from weaned piglet housing areas [32,33]. Although similar resistance determinants and shared sequence types were detected in both production stages, the cross-sectional design and seven-locus MLST do not establish persistence, transmission, or recent common ancestry. Independent introductions into the two housing areas cannot be excluded. Whole-genome sequencing combined with epidemiological linkage and longitudinal sampling would be required to investigate these possibilities. Future studies incorporating whole-genome sequencing and longitudinal sampling within individual farms would allow more detailed investigation of transmission pathways, plasmid dynamics, and evolutionary relationships among resistant isolates. Such approaches would help determine whether the similarities observed between production stages reflect clonal transmission, plasmid dissemination, or independent introductions.

This study had several limitations. Because MLST was restricted to the nine PMQR-positive isolates, findings regarding sequence-type distribution and MLST-based relationships apply only to this subset and cannot be generalized to all 40 CIP-resistant isolates or to the overall *E. coli* population. Additionally, whole-genome sequencing was not performed; therefore, additional resistance determinants or mobile genetic elements associated with FQ resistance may not have been fully captured. Although isolate selection was performed before antimicrobial susceptibility testing and balanced across farms and production stages, only one *E. coli* isolate per production stage per farm was analyzed. This sampling strategy may not fully capture within-farm strain and resistance diversity, and the observed resistance frequencies should not be interpreted as population-level prevalence or distribution within each production stage. Culture-dependent recovery and single-colony selection may have introduced selection bias, including failure to detect low-abundance resistant subpopulations. The study was not designed to achieve a prespecified level of statistical power. The limited number of farm pairs may have reduced the precision of the estimates and the ability to detect smaller differences in resistance frequencies; therefore, the comparisons should be interpreted as exploratory. Farm-level antimicrobial use and management factors were not measured. Antimicrobial exposure therefore remains a potential unmeasured confounder, and the observed difference in CIP resistance cannot be attributed to increased antimicrobial selection pressure during weaning or specific management conditions. Finally, because this was a cross-sectional study and the same animals were not followed from suckling to weaning, the observed differences between production stages cannot establish that weaning itself caused amplification or persistence of CIP-resistant *E. coli*.

## 5. Conclusions

CIP-resistant *E. coli* isolates from suckling and weaned piglet housing areas in Korea showed widespread multidrug resistance. Among all 70 *E. coli* isolates, CIP resistance was more frequent in isolates from weaned piglet housing areas than in those from suckling-piglet housing areas. QRDR mutations in *gyrA* and *parC* were detected in most CIP-resistant isolates, and PMQR determinants and additional resistance genes were identified among isolates with diverse sequence types. The detection of PMQR-positive isolates belonging to the same sequence type, particularly ST5229, in both production stages indicates that similar genetic backgrounds were present among isolates from both suckling and weaned piglet housing areas. These findings provide a basis for further investigation of production-stage differences in antimicrobial resistance and the potential value of early-life sampling in surveillance.

## Figures and Tables

**Table 1:** QRDR mutations, plasmid-mediated quinolone resistance (PMQR) genes, and fluoroquinolone MICs of 27 ciprofloxacin-resistant *Escherichia coli* isolates with detected QRDR mutations from suckling and weaned piglet housing areas in Korea.

Housing Area	Isolates	Amino Acid Change ^(1)^		PMQR Gene ^(2)^	MIC (μg/mL) ^(3)^		
		*gyrA*	*parC*		CIP	ENR	NOR
Suckling piglet	627	S83L/D87N	S80I	*qnrD*	32	128	256
housing area	689	S83L/D87N	S80I	*qnrD*	8	128	8
	672	D87N	WT	*qnrD*	1	8	1
	972	S83L	WT	*qnrS:qepA*	64	256	8
	650	S83L/D87N	S80I	-	64	>256	256
	128	S83L/D87N	S80I	-	16	>256	256
	154	S83L/D87N	S80I	-	2	256	256
	224	S83L/D87N	S80I	-	8	>256	256
	272	S83L/D87N	S80I	-	64	>256	256
	988	S83L/D87N	WT	-	32	256	256
	247	D87N	S80I	-	16	256	256
	78	D87N	S80I	-	16	256	256
	390	D87N	S80I	-	128	>256	256
Weaned piglet	109	S83L/D87N	S80I	*qnrB*	128	>256	256
housing area	652	S83L/D87N	S80I	*qnrS*	16	256	256
	575	S83L/D87N	S80I	*qnrD*	16	256	256
	691	S83L/D87N	S80I	*qnrD*	16	128	256
	316	D87N	S80I	*qnrD:aac(6′)-Ib-cr*	32	>256	256
	706	S83L/D87N	S80F	-	256	>256	256
	759	S83L/D87N	S80I	-	64	>256	256
	179	S83L/D87N	S80I	-	32	>256	256
	295	S83L/D87N	S80I	-	32	>256	256
	81	S83L/D87N	S80I	-	32	256	256
	745	S83L/D87N	S80I	-	16	128	8
	60	S83 */D87N	S80I	-	128	>256	256
	274	S83 */D87N	S80I	-	64	>256	256
	642	S83 */D87G	S80R	-	32	128	256

^(1)^ WT: wild-type; ^(2)^ -: not detected; ^(3)^ MIC: minimum inhibitory concentration; CIP: ciprofloxacin; ENR: enrofloxacin; NOR: norfloxacin. *: An asterisk denotes a stop codon in the translated sequence. For quality control, *E. coli* ATCC 25922 showed MIC values of CIP: <0.125 μg/mL, ENR: <0.125 μg/mL, NOR: 0.5 μg/mL. Only the 27 isolates with detected QRDR mutations are presented. All 40 CIP-resistant isolates were analyzed; no mutations were detected in the analyzed regions of *gyrA* and *parC* in the remaining 13 isolates.

## Data Availability

The datasets generated and/or analyzed during the current study are available from the corresponding author upon reasonable request. The datasets are not publicly available because they form part of an ongoing study and may be used in future publications.

## Abbreviations

The following abbreviations are used in this manuscript:

CIP	ciprofloxacin
CLSI	Clinical and Laboratory Standards Institute
ENR	enrofloxacin
FQ	fluoroquinolone
MIC	minimum inhibitory concentration
MLST	multilocus sequence typing
NOR	norfloxacin
PMQR	plasmid-mediated quinolone resistance
QRDR	quinolone resistance-determining region
ST	sequence type
